# Supplementary material for: Individual, but not population asymmetries, are modulated by social environment and genotype in Drosophila melanogaster
Source: Sci Rep. 2020 Mar 11;10:4480. doi: 10.1038/s41598-020-61410-7 (PMC7066193; doi:10.1038/s41598-020-61410-7)
Supplement: Supplementary file 1 — Supplementary materials. [file 41598_2020_61410_MOESM1_ESM.docx]

**Individual, but not population asymmetries, are modulated by social environment and genotype in *Drosophila melanogaster* - Supplementary materials**

Elisabetta Versace*^1,2^, Matteo Caffini^3^, Zachary Werkhoven^1^, Benjamin L. de Bivort*^1^

^1^ Department of Organismic and Evolutionary Biology and Center for Brain Science, Harvard University (USA)

^2^ Department of Biological and Experimental Psychology, Queen Mary University of London (UK)

Corresponding authors: e.versace@qmul.ac.uk; debivort@oeb.harvard.edu

**Supplementary tables**

|  | Df | F | p | ω^2^ |
| --- | --- | --- | --- | --- |
| **Context** | **3** | **3.624** | **0.0125** | 0.00222 |
| Sex | 1 | 0.423 | 0.5156 | -0.0002 |
| Strain | 4 | 0.783 | 0.5363 | -0.0002 |
| Context x Sex | 1 | 0.250 | 0.6170 | -0.0002 |
| **Context x Strain** | **12** | **2.093** | **0.0146** | 0.0037 |
| **Sex x Strain** | **4** | **3.271** | **0.0110** | 0.0025 |
| Context x Sex x Strain | 4 | 0.217 | 0.9291 | -0.0008 |
| Residuals | 3489 |  |  |  |

Supplementary Table 1. Results of the ANOVA and ω^2^ values on population-level circulating asymmetry for all conditions with Context, Sex and Strain as independent variables. Bold cells indicate significant results.

|  | Df | F | p | ω^2^ |
| --- | --- | --- | --- | --- |
| Dyad | 1 | 1.548 | 0.2135 | <0.001 |
| Sex | 1 | 2.301 | 0.1294 | 0.0004 |
| Strain | 4 | 0.724 | 0.5757 | -0.003 |
| DyadxDex | 1 | 3.751 | 0.0529 | 0.0008 |
| **Dyad x Strain** | **4** | **2.656** | **0.0313** | 0.0019 |
| **Sex x Strain** | **4** | **2.412** | **0.0470** | 0.0016 |
| Dyad x Sex x Strain | 4 | 0.372 | 0.8288 | -0.0007 |
| Residuals | 3499 |  |  |  |

Supplementary Table 2. Result of the ANOVA and ω^2^ values on individual-level circulating asymmetry for all conditions with Dyad (Single, Dyad), Sex and Strain as independent variables. Bold cells indicate significant results.

|  | Df | F | p | ω^2^ |
| --- | --- | --- | --- | --- |
| **Context** | **3** | **123.521** | **<2e-16** | **0.0847** |
| Sex | 1 | 0.031 | 0.859392 | -0.0002 |
| **Strain** | **4** | **5.565** | **0.000183** | 0.0042 |
| **Context x Sex** | **1** | **9.222** | **0.002409** | 0.0019 |
| **Context x Strain** | **12** | **35.368** | **<2e-16** | **0.0950** |
| Sex x Strain | 4 | 1.584 | 0.175736 | 0.0005 |
| **Context x Sex x Strain** | **4** | **4.098** | **0.002575** | 0.0029 |
| Residuals | 3489 |  |  |  |

Supplementary Table 3. Results of the ANOVA and ω^2^ values on individual-level circulating asymmetry for all conditions with Context, Sex and Strain as independent variables. Bold cells indicate significant results.

|  | Df | F | p | ω^2^ |
| --- | --- | --- | --- | --- |
| **Dyad** | **1** | **319.856** | **<2e-16** | **0.0753** |
| **Sex** | **1** | **12.705** | **0.000370** | 0.0028 |
| **Strain** | **4** | **5.184** | **0.000366** | 0.0040 |
| **Dyad x Sex** | **1** | **24.439** | **8.03e-07** | 0.0055 |
| **Dyad x Strain** | **4** | **43.405** | **<2e-16** | 0.0401 |
| **Sex x Strain** | **4** | **30.146** | **<2e-16** | 0.0275 |
| **Dyad x Sex x Strain** | **4** | **15.311** | **2.04e-12** | 0.0135 |
| Residuals | 3499 |  |  |  |

Supplementary Table 4. Results of the ANOVA and ω^2^ values on individual-level circulating asymmetry for all conditions with Dyad, Sex and Strain as independent variables. Bold cells indicate significant results.

|  | Df | F | p | ω^2^ |
| --- | --- | --- | --- | --- |
| Context | 3 | 0.971 | 0.406 | 0.000 |
| Sex | 1 | 0.161 | 0.688 | 0.000 |
| Strain | 4 | 0.255 | 0.907 | -0.001 |
| Context x Sex | 1 | 0.004 | 0.948 | 0.000 |
| Context x Strain | 12 | 0.537 | 0.892 | -0.002 |
| Sex x Strain | 4 | 0.774 | 0.542 | 0.000 |
| Context x Sex x Strain | 4 | 0.664 | 0.617 | 0.000 |
| Residuals | 3489 |  |  |  |

Supplementary Table 5. Results of the ANOVA on wing use population-level asymmetry for all conditions with Dyad, Sex and Strain as independent variables.

|  | Df | F | p | ω^2^ |
| --- | --- | --- | --- | --- |
| **Context** | **3** | **3.734** | **0.010769** | 0.002 |
| **Sex** | **1** | **12.943** | **0.000326** | 0.003 |
| **Strain** | **4** | **10.863** | **9.42e-09** | 0.011 |
| Context x Sex | 1 | 0.113 | 0.736724 | 0.000 |
| **Context x Strain** | **12** | **3.693** | **1.47e-05** | 0.009 |
| Sex x Strain | 4 | 0.838 | 0.500612 | 0.000 |
| **Context x Sex x Strain** | **4** | **3.856** | **0.003947** | 0.003 |
| Residuals | 3489 |  |  |  |

Supplementary Table 6. Results of the ANOVA on wing use individual-level asymmetry for all conditions with Dyad, Sex and Strain as independent variables. Bold cells indicate significant results.

**Supplementary materials 1. Velocity**

As a proxy for general activity in the testing arena, we analyzed the average velocity of flies. When comparing all contexts (FF, MM, FM and single flies) we observed a significant effect and a high ω^2^ for Context (F_3,3489_=168.932, p<2e-16, ω^2^=0.10 see Figure S1.1a) and Strain (F_4,3489_=176, p<2e-16, ω^2^=0.14, see Figure S1.1b), while the other significant main effects and interactions had a small explanatory power, see Fig. 1c and Table S1.1 for the complete results.


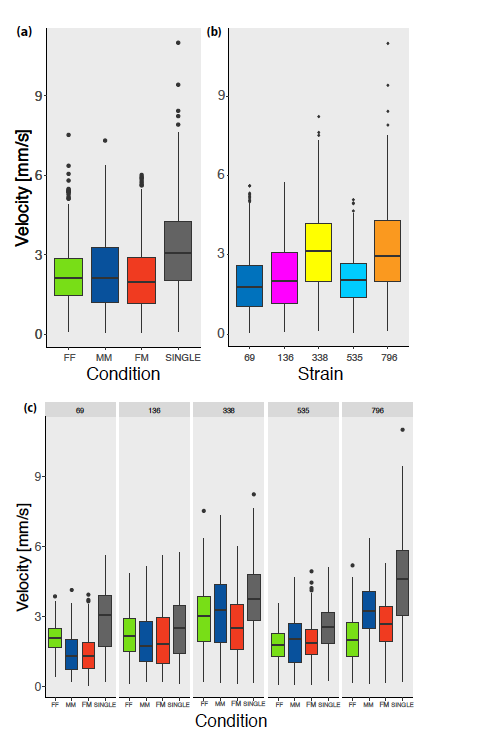


Figure S1.1. (a) Overall mean velocity by Context: dyads of two females (FF), two males (MM), one male and one female (FM), individual flies (SINGLE); (b) Overall mean velocity by Strain (c) Overall mean velocity by Context and Strain.

|  | Df | F | p | ω^2^ |
| --- | --- | --- | --- | --- |
| **Context** | **3** | **168.932** | **<2e-16** | **0.1004** |
| **Sex** | **1** | **21.267** | **4.14e-06** | 0.0040 |
| **Strain** | **4** | **176.111** | **<2e-16** | **0.1397** |
| **Context x Sex** | **1** | **14.910** | **0.000115** | 0.0028 |
| **Context x Strain** | **12** | **21.461** | **<2e-16** | 0.0489 |
| **Sex x Strain** | 4 | 2.162 | 0.070729 | 0.0009 |
| **Context x Sex x Strain** | **4** | **2.856** | **0.022328** | 0.0015 |
| Residuals | 3489 |  |  |  |

Table S1.1. Results of ANOVA and ω^2^ values on the variable Velocity for all conditions with Context, Sex and Strain as independent variables. Bold cells indicate significant results or a large portion of the variance explained by that factor.

Because Context includes dyads and single tested flies, we further explored the difference between these two nested variables. A post-hoc ANOVA showed that flies in the single context moved faster than dyads (see Figure S1.2a): F_1,3499_=487.41, p<2e-16, ω^2^=0.09, see Table S1.2 for the complete results. In contrast, an ANOVA on the dyadic contexts showed that the significant difference between contexts explained a small fraction of the variance (F_2,2500_=8.621, p<0.001, ω^2^=0.005), and that Strain was by far the strongest explanatory factor (F_4,2500_=118.946, p<2e-16, ω^2^=0.149), see Table S1.3 for the complete results. This is consistent with the finding that genotype had a bigger effect on the mean and variance of several behavioral measures than environmental treatment or the interaction of treatment and genotype ^1^. Locomotion faster in individual flies than in dyads with different sex had been previously showed ^2^, while it was not previously reported that same sex dyads moved slower than individual flies. This shows that the slower speed observed in dyads in not specifically due to inter-sexual behaviors.

|  | Df | F | p | ω^2^ |
| --- | --- | --- | --- | --- |
| **Dyad** | **1** | **487.409** | **<2e-16** | **0.0979** |
| **Sex** | **1** | **21.317** | **4.03e-06** | 0.0041 |
| **Strain** | **4** | **175.542** | **<2e-16** | **0.1406** |
| **Dyad x Sex** | **1** | **14.420** | **0.000149** | 0.0027 |
| **Dyad x Strain** | **4** | **30.585** | **<2e-16** | 0.0238 |
| **Sex x Strain** | **4** | **20.658** | **<2e-16** | 0.0158 |
| **Dyad x Sex x Strain** | **4** | **9.165** | **2.30e-07** | 0.0066 |
| Residuals | 3499 |  |  |  |

Table S1.2. Results of the ANOVA and ω^2^ values on the variable Velocity for all conditions with Dyad, Sex and Strain as independent variables. Bold cells indicate significant results or a large portion of the variance explained by that factor.

|  | Df | F | p | ω^2^ |
| --- | --- | --- | --- | --- |
| **Context** | **2** | **8.621** | **0.000186** | 0.0048 |
| Sex | 1 | 0.192 | 0.661174 | -0.0003 |
| **Strain** | **4** | **118.946** | **<2e-16** | **0.1486** |
| **Context x Strain** | **8** | **19.677** | **<2e-16** | 0.0470 |
| **Sex x Strain** | **4** | **5.761** | **0.000130** |  |
| Residuals | 2500 |  |  |  |

Table S1.3. Results of the ANOVA and ω^2^ values on the variable Velocity for dyadic contexts with Context, Sex and Strain as independent variables. Bold cells indicate significant results or a large portion of the variance explained by that factor.

A post-hoc ANOVA on the single tested flies showed that the main source of variability in Velocity was Strain (F_4,2500_=119, p<2e-16, ω^2^=0.23), while Sex had a significant effect but explained little variance (F_1,989_=3.9e-07, ω^2^=0.019) and the interaction Sex x Strain was not significant. See Table S1.4 for the complete results.

Overall, single flies consistently moved faster than flies in dyads (Figures S1.1a, S1.1c) with strong modulation by genotype, while the sex composition and social context had limited effects. Genetic variation for locomotor activity has been documented in the DGRP collection ^1,3^, including some of the strains investigated here, although other studies failed to observe significant differences between strains when comparing same sex groups ^4^. Here, we observe a significant difference between strains with a large portion of the variance explained by the factor Strain: F_3,380_=51.3, p<2e-16, ω^2^=0.282, see Figure S1.2).

|  | Df | F | p | ω^2^ |
| --- | --- | --- | --- | --- |
| **Sex** | **1** | **26.094** | **3.9e-07** | 0.0190 |
| **Strain** | **4** | **76.846** | **<2e-16** | **0.2292** |
| Sex x Strain | 4 | 0.085 | 0.987 | -0.00276 |
| Residuals | 989 |  |  |  |

Table S1.4. Results of the ANOVA and ω^2^ values on the variable Velocity for the single flies with Sex and Strain as independent variables. Bold cells indicate significant results or a large portion of the variance explained by that factor.

Considering the number of turns in a Y-maze reported by Ayroles and colleagues ^3^ as a proxy for velocity (assuming that flies circulated in the maze using the entire space), we observe a significant difference between strains with a large portion of the variance explained by the factor Strain, similarly to what we found here: F_3,380_=51.28, p<2e-16, omega-squared=0.282, see Supplementary Figure S1.2).


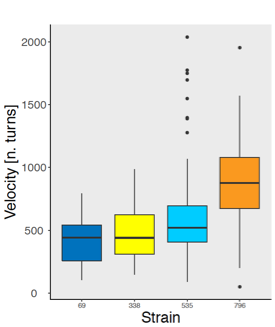


Figure S1.2. Velocity measured as number of turns in a Y-maze by Strain during two hours of observation (data from ^3^). The sample size is RAL-69=111, RAL-338=62, RAL-535=110, RAL-796=101.

Looking at time courses of Velocity for each Context and Sex (Figure S1.3), we observed that strains RAL-69, RAL-136 and RAL-535 showed roughly stable patterns over the course of each experiment, while RAL-338 and RAL-796 exhibited an increase of velocity in time, with the exception of females RAL-796 in FM dyads. An increase of locomotor activity due to starvation had been previously well described ^5–7^. It is likely that the increase of activity observed in some genotypes was a response to food deprivation, given that no food was provided in the test arena. It is clear that velocity trajectories across the experiment were modulated by both genotype and social context.


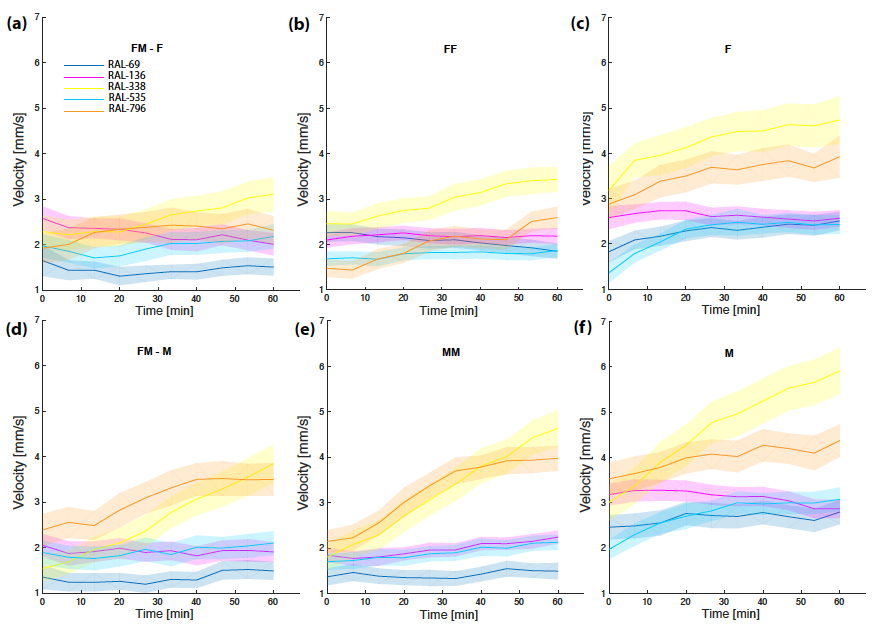


Figure S1.3. Velocity vs. time for different Strains, by Context and Sex. (a) Females in FM dyads, (b) females in FF dyads, (c) females tested individually, (d) females in FM dyads, (e) males in MM dyads, (f) males tested individually. The solid line is the mean value at each time point, and the shaded region is the standard error of the mean.

**Supplementary materials 2: Distance**

The distribution of the Distance by Strain and Context is shown in Figure S2.1. MM and FM dyads had bimodal distributions, with a “close” peak around 3.3 mm (MM= 3.20, FM=3.39 mm, Fig. S2.1c,e) and a “distant” peak around 13 mm, which is close to the distance expected for flies that are randomly positioned within the arena. FF dyads only had the distant peak at 13.06 mm (Fig. 9a). The average distance in each context ± standard deviation was: FF=12.84 mm ± 0.96, FM=11.40 mm ± 2.11, MM=12.24 mm ± 1.51.


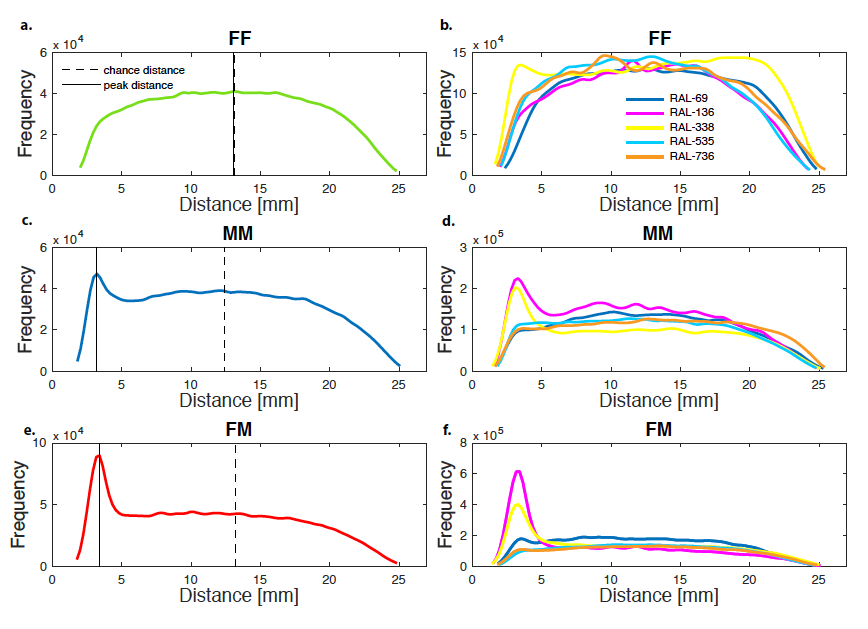


Figure S2.1. Distribution of Distance during the test in each Context and Strain (a) FF dyads, (b) FF dyads by strain, (c) MM dyads, (d) MM dyads by strain, (e) FM dyads, (f) FM dyads by strain. Dashed lines indicate the chance distance, black solid lines indicate the peak distance observed.

We assessed the effect of Context, Sex and Strain on Distance using ANOVA. We observed a significant effect of Context (F_2,1240_=114, p<2e-16, ω^2^=0.118, Figure S2.1a,c,e, Figure S2.2a) and Strain (F_4,1240_=56.7, p<2e-16, ω^2^=0.117, Figure S2.1b,d,f, S2.2b) and a significant interaction Context x Strain (F_8,1240_=26.6, ω^2^=0.107, Figure S2.1b,d,f, Figure S2.2c), while Sex and Sex x Strain were not significant, see Table S2.1 for the complete results. The significant Strain x Context interaction mainly reflects the fact that strains RAL-136 and RAL-338 had clear close peaks at about 3 mm in MM and FM dyads, while other strains lacked or showed reduced peaks there.

|  | Df | F | p | ω^2^ |
| --- | --- | --- | --- | --- |
| **Context** | **2** | **113.968** | **<2e-16** | **0.1182** |
| Sex | 1 | 0.745 | 0.388 | -0.0001 |
| **Strain** | **4** | **56.686** | **<2e-16** | **0.1165** |
| **Context x Strain** | **8** | **26.615** | **<2e-16** | **0.1072** |
| Sex x Strain | 4 | 0.663 | 0.618 | -0.0007 |
| Residuals | 1240 |  |  |  |

Table S2.1. Results of the ANOVA and ω^2^ values on Distance with Context, Sex and Strain as independent variables. Bold cells indicate significant results.

**
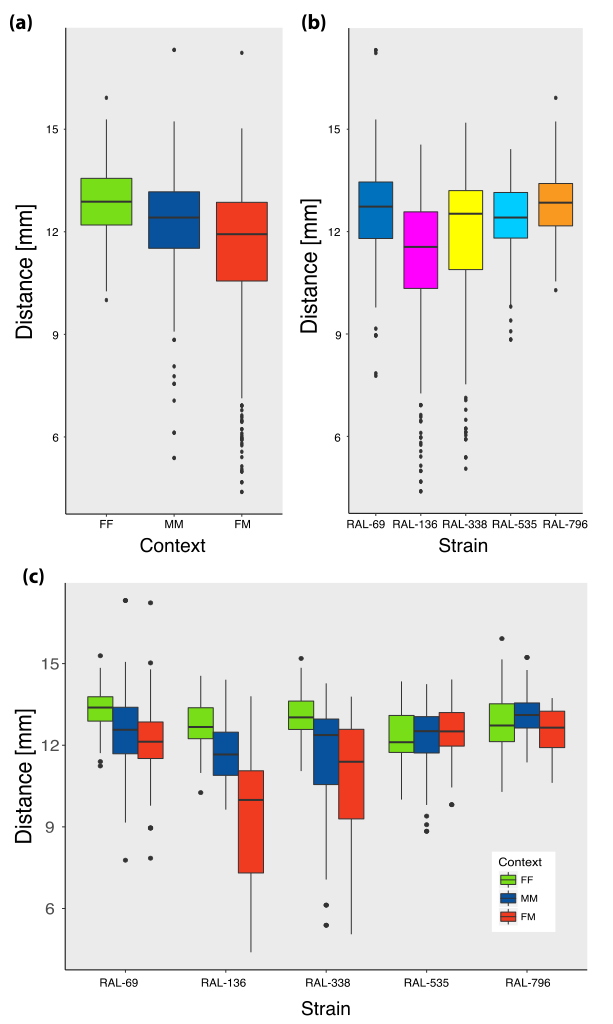
**

Figure S2.2. (a) Distance between flies by Context (FF, MM, FM), (b) by Strain (c) and by Context and Strain.

Post hoc ANOVAs showed significant effects of Context, but the effect size differed between comparisons: FF-FM (F_1,856_=159, p<2e-16, ω^2^=0.155), FF-MM (F_1,801_=53.1, p=7.7e-13, ω^2^=0.061), FM-MM (F_1,857_=39.1, p=6.33e-10, ω^2^=0.042). As expected from the interactions of courtship and mating, flies in the heterosexual dyads were closer than flies in the other contexts (Fig. S2.2a). Dyads with two females (FF) were significantly further than other dyads (Fig. S2.2a), about as far as expected from non-interacting flies. The fact that FM and MM dyads stayed closer than FF dyads, together with previous observations about intersex interactions (e.g., ^8^), is consistent with the males initiating courtship interactions in this species.

The distance between flies was clearly modulated by the social environment, genotype and their interaction. Flies in the FM and MM contexts stayed closer than expected by chance but only some genotypes (RAL-69, RAL-136 and RAL-338) had a distinct peak distance at around 3 mm. Previous studies have shown that flies communicate not only through chemosensory stimuli but also using mechanosensory interactions that induce flies to move in space ^4,9^. For this reason, genetic variation for distance has implications for the amount of information transferred between flies and their experience with the environment.

**References**

1. Akhund-Zade, J., Ho, S., O’Leary, C. & de Bivort, B. The effect of environmental enrichment n behavioral variability depends on genotype, behavior, and type of enrichment. *biorxiv* (2019).

2. Klibaite, U. *et al.* An unsupervised method for quantifying the behavior of paired animals. *Phys. Biol.* **14**, (2017).

3. Ayroles, J. F. *et al.* Behavioral idiosyncrasy reveals genetic control of phenotypic variability. *Proc. Natl. Acad. Sci.* **112**, 201503830 (2015).

4. Schneider, J., Atallah, J. & Levine, J. D. One, Two, and Many. A Perspective on Whar Groups of *Drosophila melanogaster* Can Tell Us About Social Dynamics. in *Advances in Genetics* **77**, 59–78 (Elsevier Inc., 2012).

5. Bell, W. J., Cathy, T., Roggero, R. J., Kipp, L. R. & Tobin, T. R. Sucrose-stimulated searching behaviour of *Drosophila melanogaster* in a uniform habitat: modulation by period of deprivation. *Anim. Behav.* **33**, 436–448 (1985).

6. Connolly, K. J. Locomotor Activity in *Drosophila* as a Function of Food Deprivation. *Nature* **209**, 224 (1966).

7. Knoppien, P., van Der Pers, J. & van Delden, W. Quantification of locomotion and the effect of food deprivation on locomotor activity in *Drosophila*. *J. Insect Behav.* **13**, 27–43 (2000).

8. Gowaty, P. A., Steinichen, R. & Anderson, W. W. Indiscriminate Females and Choosy Males: Within- and Between-Species Variation in *Drosophila*. *Evolution.* **57**, 2037–2045 (2003).

9. Ramdya, P. *et al.* Mechanosensory interactions drive collective behaviour in *Drosophila*. *Nature* **519**, 233–236 (2014).
